# Perceptions of Fungal Contamination in Hospital Humidifiers Among Healthcare Providers in Jordan: A Cross-Sectional Study

**DOI:** 10.7759/cureus.88721

**Published:** 2025-07-25

**Authors:** Zaina B Rawashdeh, Mohammad A Al-Shalalfeh, Abdulrahman M Alsarhan, Leen O Sabbah, Khaled Kettaneh, Farah M Shahin, Basil G Daradkeh, Nour A Alaqqad, Subhi M Nassar, Nada M Abujbara

**Affiliations:** 1 Faculty of Medicine, University of Jordan, Amman, JOR; 2 Faculty of Medicine, Mutah University, Irbid, JOR; 3 Medicine, Al-Balqa Applied University, Amman, JOR; 4 General Practice, Jordan University of Science and Technology, Irbid, JOR; 5 General Practice, Um Zweitena Private Clinic, Irbid, JOR

**Keywords:** cleaning protocols, fungal contamination, hospital-acquired infections, humidifiers, infection control, intensive care units (icus), jordan

## Abstract

Background: Hospital-acquired infections remain a critical concern globally. Fungal contamination of reusable hospital humidifiers, especially in intensive care units (ICUs), represents an under-recognized threat. These devices, if improperly maintained, may become reservoirs for opportunistic pathogens such as Aspergillus and Fusarium, thereby compromising patient safety.

Objective: The objective of this study is to explore the perceptions of fungal contamination in hospital humidifiers across Jordan and describe associated disinfection practices, protocol presence, and staff awareness, with the aim of informing targeted preventive strategies.

Methods: A cross-sectional observational study was conducted from May 1 to June 1, 2025, using an anonymous, self-administered online questionnaire distributed via hospital email lists and official WhatsApp groups. Eligible participants included physicians, nurses, residents, medical students, and infection control staff from departments that regularly use humidifiers (e.g., ICUs, pediatrics, internal medicine, emergency medicine). The survey collected data on disinfection routines, protocol availability, and awareness of fungal risks. In total, 50 valid responses were analyzed. Descriptive statistics were used, and Chi-square and Fisher’s Exact Tests were applied to assess associations. However, the findings are limited by the reliance on self-reported data, the absence of microbiological validation, limited details on humidifier types and maintenance, potential departmental confounding, and the small convenience sample (n=50), which may affect generalizability. Ethical approval was not required under local guidelines, given the minimal-risk nature of the study.

Results: Of the 50 valid responses, self-reported data showed that 29 participants (58%) observed previous fungal contamination in their department’s humidifiers. Contamination was reported in all humidifiers located in ICUs (n = 17, 100%) and general wards (n = 2, 100%). A statistically significant association was observed between cleaning frequency and contamination (p = 0.0034), with higher contamination among frequently cleaned devices. Geographic disparities were also noted, with Amman showing a 100% (n = 19) contamination rate, compared to 7.7% (n = 1) in Irbid (p < 0.001). While 44 (88%) participants were aware of fungal risks, only 27 (54%) reported having written cleaning protocols. Results are limited by reliance on unverified staff reports. Therefore, contamination rates were based on participant perception and may be influenced by department-specific practices or equipment use frequency.

Conclusion: Fungal contamination in hospital humidifiers is widespread, particularly in ICUs. The paradoxical association between frequent cleaning and higher contamination may reflect increased usage and confounding by department type. These findings highlight the urgent need for standardized protocols, microbiological monitoring, and staff training to mitigate nosocomial fungal infections in Jordan.

## Introduction

Hospital-acquired infections pose a significant challenge to patient safety and healthcare outcomes worldwide. Devices commonly used in hospitals, such as humidifiers in oxygen delivery systems and neonatal care, can create environments that encourage fungal growth. This overlooked reservoir is a potential risk to patient safety, silently introducing pathogens into clinical settings. While international studies suggest that humidifiers may harbor fungal pathogens, Jordan-specific data are limited. Accordingly, when sanitary cleaning and maintenance are inadequate, these apparatuses become contaminated and create conditions that facilitate infection via inhalation of aerosols from contaminated reservoirs, endangering vulnerable patients in ICUs, neonatal ICUs, and respiratory wards [1].

Reusable hospital humidifiers employed in oxygen therapy may harbor fungal spores in some settings, creating a moist environment conducive to fungal proliferation [2]. One study reported pervasive microbial contamination in every tested reusable device, including fungal species with pathogenic potential, while disposable prefilled humidifiers showed substantially lower contamination levels, primarily during the initial weeks of use [3]. Furthermore, outbreaks linked to water-containing hospital devices underscore the risk of aerosolized fungi being transmitted to patients, particularly in intensive care and neonatal settings, where immune defenses are compromised and fungal infections are severe [4]. 

Fungal species such as Aspergillus were recovered from 70% of hospital water samples in a three-year study of a bone marrow transplant unit's distribution system. A total of 109 water samples yielded Aspergillus species [5]. The species of Aspergillus found were all recognized opportunistic pathogens and included A. niger (77%), A. fumigatus (11%), A. terreus (9%), and A. flavus (3%). All the Paecilomyces species found in 44 water samples were Plilacinus [5]. Three of 14 Fusarium species recovered from water samples were identified to the species level. All three were F. solani (known pathogens). The mean concentration ± SD of molds was 9-fold higher in the water tanks than in the municipal water: mean 11.4 ± 14.0 versus. 1.3 ± 1.7 CFU/L, respectively (P< 0.01), raising the possibility that hospital water systems, including humidifiers, may serve as potential reservoirs for airborne fungi [5]. With the exception of Chaetomium species, all molds recovered from municipal water were also present in the water tank. Penicillium species and Aspergillus species were the two most common molds recovered from municipal water and hospital water. Penicillium species were present in 33%, 42%, and 43% of the samples of municipal water, water storage tanks, and water from patient care areas, respectively, whereas Aspergillus species were recovered in 33%, 55%, and 21%, respectively, of water samples obtained from these same sites [6]. Moreover, a 2013 study reported that over 75% of reusable oxygen humidifiers were contaminated with various fungal species, such as Aspergillus and Fusarium, underscoring the heightened risk posed by inadequate cleaning and reuse of these devices in health care settings [6].

As hospital humidifiers are increasingly recognized as a reservoir of fungal contamination, data remain scarce within Jordanian healthcare settings [7]. Despite existing infection control activities, published data on fungal contamination and routine surveillance in Jordanian hospitals remain insufficient, mostly owing to a lack of standardized guidelines and resource constraints. A study conducted in Zarqa, Jordan, identified Aspergillus, Penicillium, Rhizopus, and Alternaria species in indoor air samples from intensive care units (ICUs) and neonatal wards, emphasizing the occurrence of fungal pathogens in hospital environments [8]. To address this gap, we conducted a cross-sectional study using self-administered questionnaires distributed to healthcare providers, evaluating both contamination prevalence and infection control practices. Given the lack of national data on humidifier maintenance practices, this study first aimed to capture frontline healthcare workers' experiences through a survey. This approach identifies gaps to guide future microbiological studies.

## Materials and methods

This cross-sectional observational study was conducted between May 1 and June 1, 2025, using an anonymous, self-administered online questionnaire. The study targeted healthcare professionals working in both public and private hospitals across Jordan, including major care centers in Amman and regional hospitals in Irbid, Zarqa, and others. Clinical departments represented included internal medicine, pediatrics, emergency medicine, and ICUs, where respiratory humidifiers are commonly used.

Participants were invited through digital platforms, including hospital mailing lists and official WhatsApp groups. The questionnaire was developed using Google Forms and required approximately five minutes to complete. No physical materials, laboratory tests, or reagents were used, and all data were collected electronically.

The questionnaire included items on hospital department, presence and disinfection of humidifiers, awareness of fungal contamination, and knowledge of associated infections. Participants were also asked whether fungal contamination had been observed in their department and, if so, which fungal infections were identified. Fungal contamination data were based on self-reported observations without laboratory confirmation.

Key terms were operationally defined within the questionnaire to ensure consistent interpretation among participants. Frequent cleaning was explicitly defined as disinfection occurring daily, while the presence of protocols referred to the existence of written departmental guidance for humidifier cleaning procedures. The construct of awareness of contamination was defined as respondents' understanding that improperly maintained humidifiers could serve as reservoirs for fungal growth. These definitions were strategically positioned alongside their corresponding survey items to provide immediate clarification during questionnaire completion.

This study did not involve patients, clinical interventions, or the collection of any personal identifying data. Informed consent was obtained electronically. Ethical approval was waived based on institutional minimal-risk research guidelines.

Participants

Inclusion criteria were healthcare professionals aged 18 years or older working in a Jordanian hospital at the time of the survey. Eligible roles included physicians, residents, medical students, nurses, and infection control staff. Respondents not affiliated with hospitals or who indicated ineligibility in the questionnaire were excluded from the final analysis. The questionnaire distribution discouraged multiple submissions. Eligibility was confirmed via screening questions about hospital/ department affiliation. A total of 50 valid and complete responses were included in the analysis. Convenience sampling was used. Invitations were distributed via hospital mailing lists, official WhatsApp groups, and administrative email announcements. The message explained the purpose of the study, confirmed anonymity, and included a secure Google Forms link. Two reminder messages were sent during the data collection period to encourage participation. The invitation clearly asked participants to submit only one response.

Data measurement

The primary outcome was whether participants reported fungal contamination in hospital humidifiers (Yes/No). Exposure variables included disinfection (categorized as daily, weekly, monthly, or never) and the presence of written cleaning protocols. Confounding factors considered were hospital department type and participant awareness of contamination risks. Additional variables included the professional role, hospital governorate, responsibility for humidifier maintenance, and knowledge of fungal infections linked to humidifiers (e.g., pneumonia, bloodstream infections, fungal sinusitis). The need for increased staff awareness or training was also assessed through a direct yes/no question.

Statistical analysis

Descriptive statistics were used to summarize the data. Categorical variables were reported as frequencies and percentages. Associations between reported fungal contamination and categorical predictors, such as cleaning, presence of written protocols, and department type, were evaluated using chi-square and Fisher's exact tests. A p-value <0.05 was considered statistically significant. Incomplete or duplicate responses were excluded from the final analysis. All statistical analyses were conducted using Microsoft Excel (version 365; Microsoft Corporation, Redmond, USA). However, we limited the analysis to univariate comparisons without adjustment for potential confounders. Assumptions for chi-square tests (i.e., expected frequencies ≥5) were verified before application. No missing data were imputed; incomplete responses were not present. No adjustments for multiple comparisons were made, as the study was exploratory in nature.

## Results

**Figure 1:**
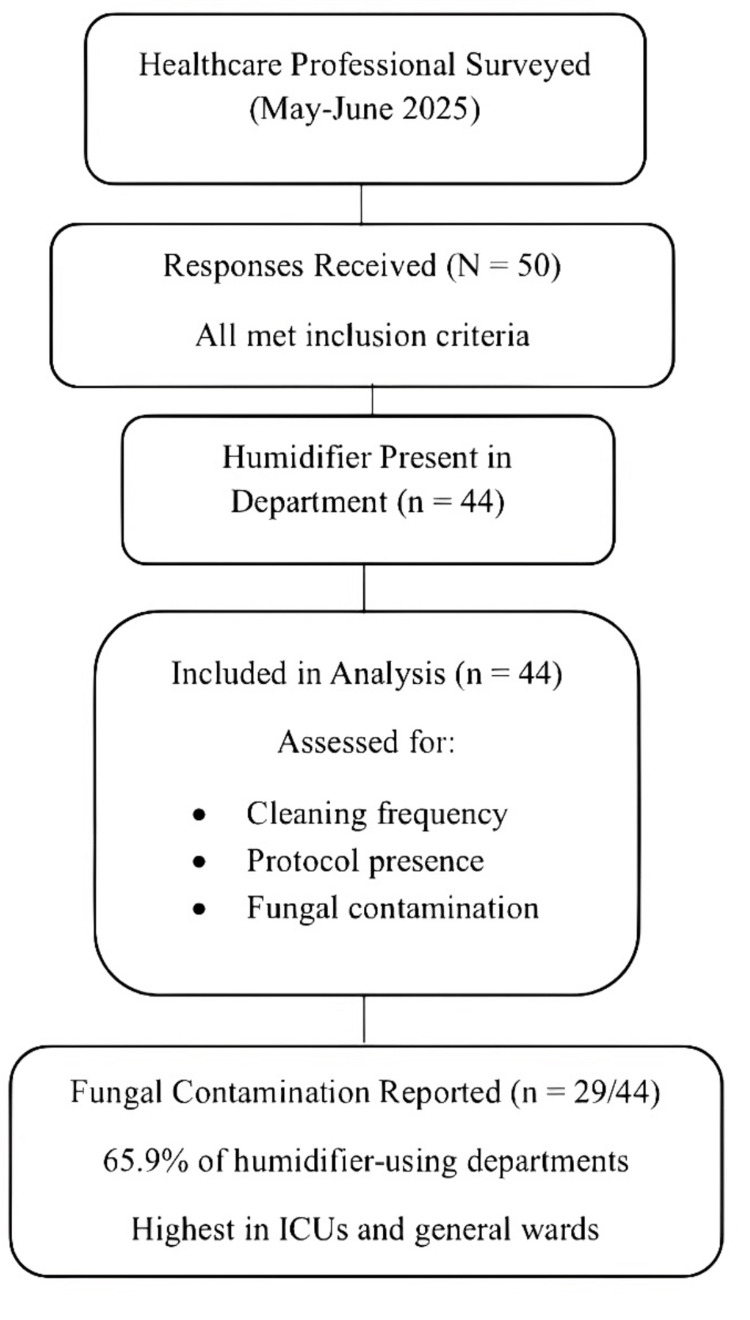
Flow Diagram of Participant Inclusion and Analysis of Humidifier Contamination Flow diagram depicting the inclusion and exclusion of survey participants based on humidifier use and response completeness.

Between May and June 2025, a total of 50 healthcare professionals completed the online survey assessing humidifier use and fungal contamination in hospital settings across Jordan. After excluding six incomplete responses, 50 fully completed questionnaires were analyzed. All participants met the inclusion criteria based on their professional affiliation with hospital departments. Data collection was based on self-reported observations and experiences. The survey captured responses from a range of hospitals and cities, providing a geographically diverse dataset. Contamination rates were based on self-reported perceptions by participants and not on direct environmental testing. Among respondents, 44 (88%) reported the presence of humidifiers in their departments and were included in the contamination and cleaning analysis. The remaining six respondents (12%), who reported no humidifier use in their clinical area, were excluded from this portion of the analysis (Figure 1).

**Figure 2:**
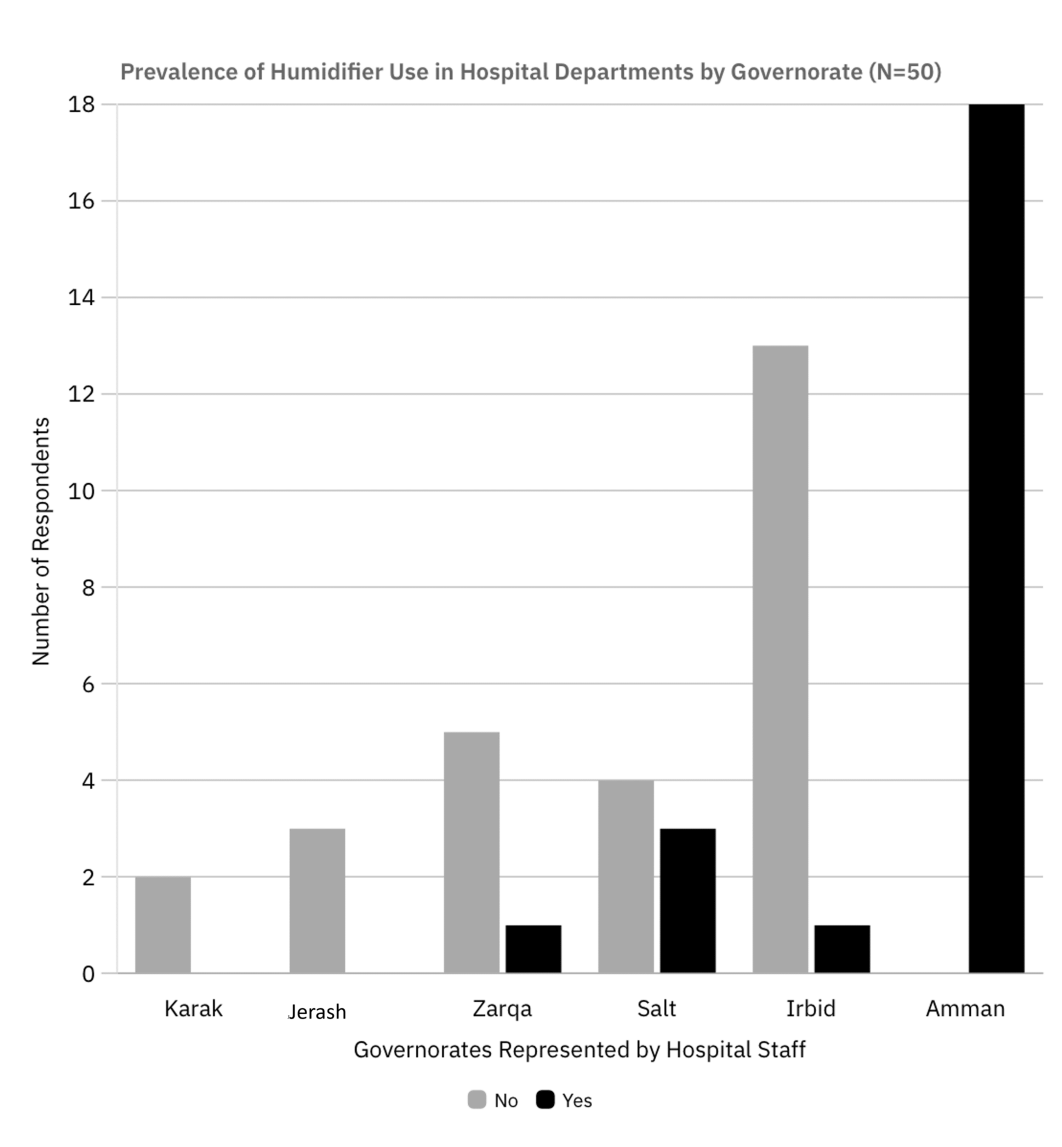
Regional Variation in the Prevalence of Humidifier Use Among Participating Hospital Departments Bar chart showing the percentage of hospital departments in each governorate reporting the use of humidifiers. Data are based on self-reported observations.

Respondents included nurses (n = 14, 28%), resident doctors (n = 12, 24%), medical students (n = 11, 22%), attending physicians (n = 7, 14%), and technicians (n = 6, 12%). The majority worked in ICUs (n = 17, 34%) and pediatric departments (n = 14, 28%), followed by operating rooms (n = 8, 16%), emergency departments (n = 4, 8%), and general wards (n = 2, 4%). Participants not affiliated with any clinical department accounted for (n = 5, 10%). Geographically, participants were primarily based in Amman (n = 19, 38%) and Irbid (n = 13, 26%), with additional representation from Salt (n = 7, 14%), Zarqa (n = 6, 12%), Jerash (n = 3, 6%), and Karak (n = 2, 4%). The prevalence of humidifier use across participating departments was evaluated by region, revealing near-universal adoption among surveyed hospitals (Figure 2). In Amman, all respondents (n = 19, 100%) reported the presence of humidifiers. In contrast, a minority of respondents in Irbid (n = 13), Zarqa (n = 6), Jerash (n = 3), Salt (n = 7), and Karak (n = 2) indicated that no humidifier was present in their work area. This observation complements the contamination findings by suggesting targeted areas for preventive interventions.

Cleaning practices varied substantially across departments. Daily cleaning was reported by 17 participants (34%), monthly by 16 participants (32%), weekly by 11 participants (22%), rarely by five participants (10%), and never by one participant (2%). Responsibility for cleaning was typically assigned to nurses (n = 16, 32%), housekeeping staff (n = 15, 30%), or infection control units (n = 14, 28%), while five participants (10%) were unsure.

Written protocols for humidifier disinfection were reportedly present in 27 participants' (54%) departments, absent in 16 (32%) participants' departments, and unknown to seven (14%) respondents. None of the departments conducted routine environmental monitoring (e.g., humidity or temperature tracking), and details regarding device age or brand were not collected. Overall, (n = 44, 88%) of participants were aware that hospital humidifiers could become contaminated with fungi. Among participants in departments with written protocols (n = 27), 22 (81.5%) reported awareness and five (18.5%) did not. Among those without protocols (n = 16), 13 (81.3%) were aware and three (18.7%) were not. Of those unsure (n = 7), five (71.4%) were aware and two (28.6%) were not. Nearly all participants (n = 49, 98%) expressed that additional training on humidifier hygiene and fungal contamination was necessary (Table 1).

**Table 1:** Association Between Staff Awareness of Fungal Contamination and Written Disinfection Protocols for Humidifiers Values are expressed as N (%). This table compares awareness of fungal contamination risk among participants from departments with and without written disinfection protocols. No inferential statistical test was applied.

Written Disinfection Protocols	Aware of Fungal Risk (n %)	Not Aware of Fungal Risk (n %)	Total (n %)
Yes	22 (81.5%)	5 (18.5%)	27 (54%)
No	13 (81.3%)	3 (18.7%)	16 (32%)
Not sure	5 (71.4%)	2 (28.6%)	7 (14%)
Total	40 (80%)	10 (20%)	50 (100%)

Out of the 50 hospital humidifiers discussed, 29 (58%) were reported to have tested positive for fungal contamination in prior laboratory evaluations, as recalled by staff. While ICU staff reported universal contamination (100%, n=17), this finding may reflect both the department's higher device utilization and increased staff vigilance rather than exclusively indicating greater contamination risk, and general ward (n = 2, 100%) humidifiers, while only 10 of 31 (32.3%) humidifiers in other departments showed contamination. This association between department type and contamination was statistically significant (χ² = 8.76, p = 0.003) (Table 2).

**Table 2:** Distribution of Fungal Contamination by Hospital Department Values are expressed as N (%). This table shows the number and percentage of departments reporting fungal contamination by type. Statistical analysis was performed using the chi-square test. A p-value < 0.05 was considered statistically significant.

Department	Total (N)	Contamination Observed (N, %)	Test Used	Test Statistic (X)	p-value
ICU	17	17 (100.0%)	N/A	N/A	N/A
General Ward	2	2 (100.0%)	N/A	N/A	N/A
Other	31	10 (32.3%)	Chi-square	8.76	0.003

**Figure 3:**
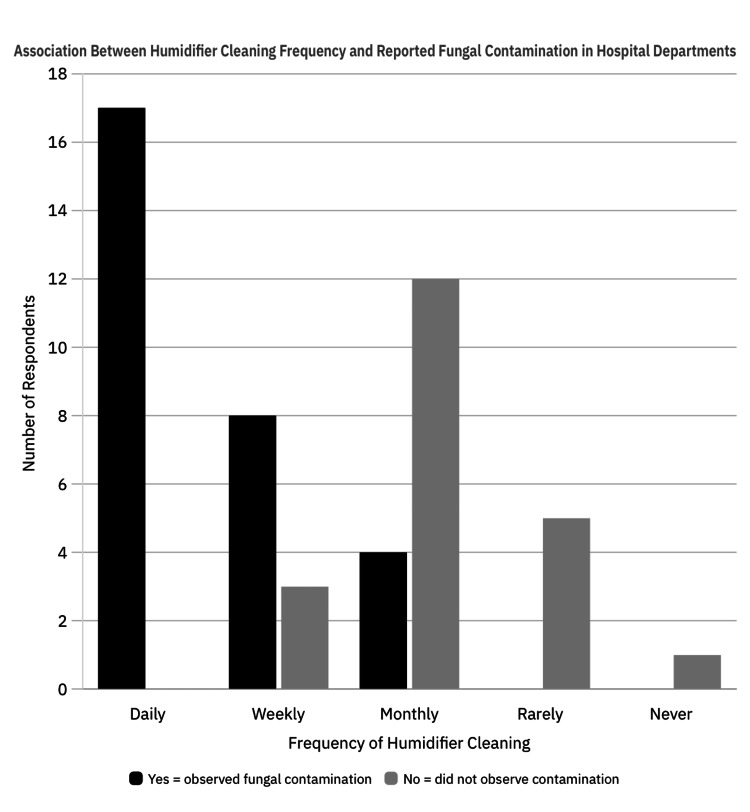
Impact of Cleaning Frequency on Detection of Fungal Contamination in Hospital Humidifiers Comparison of reported fungal contamination across different cleaning frequencies. Values are expressed as N (%). Statistical analysis was performed using Fisher’s exact test. A p-value < 0.05 was considered statistically significant.

Among respondents from departments where humidifiers were cleaned regularly (daily, weekly, or monthly; n=44), 29 (65.9%) reported having seen or heard of fungal contamination. In contrast, none of the six respondents from departments where humidifiers were rarely or never cleaned reported such observations (Figure 3). Fisher’s exact test showed a statistically significant difference (p = 0.0034) and likely reflects confounding by department type. High-use areas such as ICUs may both require more frequent cleaning and experience more frequent contamination due to constant humidifier use. 

In terms of geographic variation, 100% of respondents from Amman (n = 19) reported fungal contamination, compared to just one out of 13 (7.7%) in Irbid. In other governorates, 9 out of 18 (50%) reported contamination. The association between governorate and contamination was highly significant (χ² = 27.4, p < 0.001) (Table 3).

**Table 3:** Distribution of Fungal Contamination by Geographic Region Values are expressed as N (%). This table summarizes fungal contamination reports by governorate. Statistical analysis was performed using the chi-square test. A p-value < 0.05 was considered statistically significant.

Region	Total (N)	Contamination Observed (N, %)	Test Used	Test Statistic (X)	p-value
Amman	19	19 (100.0%)	N/A	N/A	N/A
Irbid	13	1 (7.7%)	N/A	N/A	N/A
Others	18	9 (50.0%)	Chi-square	27.4	<0.001

## Discussion

This study revealed a high prevalence of self-reported fungal contamination in reusable hospital humidifiers across Jordan, with all ICU humidifiers being contaminated. These findings are consistent with global evidence suggesting that humidifiers, especially in high-dependency settings, can serve as potential reservoirs for airborne fungal pathogens such as Aspergillus spp. and Candida spp., which are major contributors to healthcare-associated infections, particularly among immunocompromised patients [9,10].

A statistically significant association was observed between frequent cleaning and reported contamination. This paradox likely reflects confounding due to more intensive humidifier use in ICUs and high-acuity departments, where the risk of contamination increases with frequency of use, despite cleaning routines. Similar findings have been documented in studies where cleaning frequency alone did not correlate with reduced contamination unless paired with standardized disinfection protocols and staff training [11]. Regional variation was also pronounced, with all respondents from Amman reporting contamination, compared to only one (7.7%) from Irbid. These differences may reflect variable infection control practices, hospital capacities, or awareness levels. Amman’s large tertiary hospitals often manage a high patient load from across the country, which may increase exposure risks and reporting frequency [12].

Although most respondents reported routine cleaning practices and nearly half reported having disinfection protocols, one-third of departments lacked written protocols, and none conducted routine environmental monitoring. Additionally, cleaning responsibilities were primarily assigned to nurses and housekeeping staff, groups that may not always receive adequate training in sterilization procedures. These gaps may compromise decontamination efficacy and contribute to persistent contamination rates.

A key strength of this study is its inclusion of diverse hospital settings across Jordan, allowing for insights into different cleaning practices and contamination rates among various regions in Jordan. However, limitations include reliance on self-reported data without microbiological confirmation, potential confounding by department type, and lack of information on humidifier models or maintenance quality. Most importantly, the relatively low sample size limits the representativeness of the findings and reduces the statistical power of the analysis, especially given the nationwide scope of the study. Future research should include direct environmental sampling, fungal species identification, and longitudinal assessment of protocol adherence and staff training to better define and mitigate contamination risks.

Overall, the findings underscore the likelihood that hospital humidifiers are an underrecognized but preventable source of nosocomial fungal infections. Addressing this risk requires a multifaceted strategy, including protocol standardization, dedicated staff training, and microbiological surveillance. The insights gained from this study align with international recommendations emphasizing preventive environmental hygiene as a cornerstone of infection control [13].

## Conclusions

This nationwide cross-sectional study reveals a high prevalence of self-reported fungal contamination in reusable hospital humidifiers across Jordan, particularly in intensive care and general ward settings. Despite frequent cleaning, persistent contamination suggests deficiencies in disinfection protocols, staff training, and environmental oversight. The observed paradox between increased cleaning frequency and higher contamination likely reflects confounding by department type and usage intensity. Geographic disparities, such as higher contamination rates in Amman compared to Irbid, highlight inconsistencies in infection control implementation across regions. While findings are based on self-reported data, the consistency of responses supports their credibility. These results underscore the need for standardized cleaning protocols, routine staff education, and microbiological monitoring. Future research should include environmental sampling and species-level fungal identification to better inform infection control strategies and reduce nosocomial risks.

## Appendices

Appendix A

Survey Questionnaire: Fungal Contamination in Hospital Humidifiers in Jordan

**Table 4:** Structured Survey Questionnaire

Section	Question	Response Options
1. General Information	What is your current role?	○ Medical student ○ Resident doctor ○ Attending physician ○ Nurse ○ Technician ○ Other: ________
	Which hospital department do you currently work in?	○ Intensive Care Unit (ICU) ○ General ward ○ Pediatrics ○ Operating room ○ Emergency department ○ I’m not currently working in a clinical department ○ Other: ________
	Which Governorate in Jordan do you work in?	○ Amman ○ Irbid ○ Zarqa ○ Salt ○ Jerash ○ Karak ○ Other: ________
2. Humidifier Use and Cleaning	Are humidifiers used in your hospital department?	○ Yes ○ No ○ I don’t know
	How often are the humidifiers cleaned or disinfected?	○ Daily ○ Weekly ○ Monthly ○ Rarely ○ Never
	Who is primarily responsible for cleaning the humidifiers?	○ Nurses ○ Housekeeping staff ○ Infection control unit ○ I don’t know ○ Not applicable
3. Awareness and Risk	Are you aware that hospital humidifiers can become contaminated with fungi?	○ Yes ○ No
	Have you ever seen or heard of fungal growth in a humidifier in your hospital?	○ Yes ○ No
	What types of infections can result from fungal contamination in humidifiers?	□ Pneumonia □ Bloodstream infections □ Fungal sinusitis □ Skin/wound infections □ None
	Does your hospital have written protocols for cleaning humidifiers?	○ Yes ○ No ○ I’m not sure
	How serious is the risk of fungal contamination in hospital humidifiers?	○ Very serious ○ Somewhat serious ○ Not serious
	Do you believe more awareness or training is needed for hospital staff about this issue?	○ Yes ○ No

Appendix B

Consent Form

Purpose of the Study

You are invited to take part in a research study conducted by the Faculty of Medicine at the University of Jordan. This study aims to assess healthcare workers’ awareness and practices regarding fungal contamination in hospital humidifiers.

Participation Details

You will be asked to complete a short, anonymous questionnaire. The survey takes approximately 5 minutes to complete and does not collect any personal or identifying information.

Risks and Benefits

There are no expected risks from participating. While there are no direct personal benefits, your responses may help improve infection control practices in Jordanian hospitals.

Confidentiality

All responses are anonymous and will remain confidential. Data will be securely stored and used only for research purposes by the study team.

Voluntary Participation

Your participation is entirely voluntary. You may decline or stop at any time without penalty. Completion of the survey implies your consent to participate.

## References

[1] de la Fuente-Sancho I, Romeu-Bordas Ó, Fernández-Aedo I, Ballesteros-Peña S (2019). Microbiological contamination in high- and low-flow oxygen humidifiers: a systematic review. Med Intensiva.

[2] Nakipoglu Y, Erturan Z, Buyukbaba-Boral O, Aksozek A, Aydin S, Derbentli S (2005). Evaluation of the contaminant organisms of humidifier reservoir water and investigation of the source of contamination in a university hospital in Turkey. Am J Infect Control.

[3] La Fauci V, Costa GB, Facciolà A, Conti A, Riso R, Squeri R (2017). Humidifiers for oxygen therapy: what risk for reusable and disposable devices?. J Prev Med Hyg.

[4] Yiek WK, Coenen O, Nillesen M, van Ingen J, Bowles E, Tostmann A (2021). Outbreaks of healthcare-associated infections linked to water-containing hospital equipment: a literature review. Antimicrob Resist Infect Control.

[5] Anaissie EJ, Stratton SL, Dignani MC (2003). Pathogenic molds (including Aspergillus species) in hospital water distribution systems: a 3-year prospective study and clinical implications for patients with hematologic malignancies. Blood.

[6] Jadhav S, Sahasrabudhe T, Kalley V, Gandham N (2013). The microbial colonization profile of respiratory devices and the significance of the role of disinfection: a blinded study. J Clin Diagn Res.

[7] Abu-Jeyyab M, Qura'an B, Alrosan S, Al Mse'adeen M (2023). Infection control in hospitals of Jordan: challenges and opportunities. Cureus.

[8] Qudiesat K, Abu‑Elteen K, Elkarmi A, Hamad M, Abussaud M (2009). Assessment of airborne pathogens in healthcare settings. Afr J Microbiol Res.

[9] VandenBergh MF, Verweij PE, Voss A (1999). Epidemiology of nosocomial fungal infections: invasive aspergillosis and the environment. Diagn Microbiol Infect Dis.

[10] Sautour M, Sixt N, Dalle F (2007). Prospective survey of indoor fungal contamination in hospital during a period of building construction. J Hosp Infect.

[11] Mitchell BG, Hall L, White N (2019). An environmental cleaning bundle and health-care-associated infections in hospitals (REACH): a multicentre, randomised trial. Lancet Infect Dis.

[12] Al-Rawajfah OM, Hweidi IM, Alkhalaileh M, Khader YS, Alshboul SA (2013). Compliance of Jordanian registered nurses with infection control guidelines: a national population-based study. Am J Infect Control.

[13] Carling PC (2016). Optimizing health care environmental hygiene. Infect Dis Clin North Am.

